# IRE1α RIDD activity induced under ER stress drives neuronal death by the degradation of *14-3-3 θ* mRNA in cortical neurons during glucose deprivation

**DOI:** 10.1038/s41420-021-00518-9

**Published:** 2021-06-03

**Authors:** Juan Carlos Gómora-García, Cristian Gerónimo-Olvera, Xochitl Pérez-Martínez, Lourdes Massieu

**Affiliations:** 1 Departamento de Neuropatología Molecular, División de Neurociencias, Instituto de Fisiología Celular, Universidad Nacional Autónoma de México, CP 04510 Ciudad de México, México; 2 Departamento de Genética Molecular, División de Investigación Básica, Instituto de Fisiología Celular, Universidad Nacional Autónoma de México, CP 04510 Ciudad de México, México; 3 Present Address: Center for Integrative Biology, Faculty of Sciences, Universidad Mayor, Santiago, Chile

**Keywords:** Cell death in the nervous system, Stress signalling, Molecular neuroscience

## Abstract

Altered protein homeostasis is associated with neurodegenerative diseases and acute brain injury induced under energy depletion conditions such as ischemia. The accumulation of damaged or unfolded proteins triggers the unfolded protein response (UPR), which can act as a homeostatic response or lead to cell death. However, the factors involved in turning and adaptive response into a cell death mechanism are still not well understood. Several mechanisms leading to brain injury induced by severe hypoglycemia have been described but the contribution of the UPR has been poorly studied. Cell responses triggered during both the hypoglycemia and the glucose reinfusion periods can contribute to neuronal death. Therefore, we have investigated the activation dynamics of the PERK and the IRE1α branches of the UPR and their contribution to neuronal death in a model of glucose deprivation (GD) and glucose reintroduction (GR) in cortical neurons. Results show a rapid activation of the PERK/p-eIF2α/ATF4 pathway leading to protein synthesis inhibition during GD, which contributes to neuronal adaptation, however, sustained blockade of protein synthesis during GR promotes neuronal death. On the other hand, IRE1α activation occurs early during GD due to its interaction with BAK/BAX, while ASK1 is recruited to IRE1α activation complex during GR promoting the nuclear translocation of JNK and the upregulation of *Chop*. Most importantly, results show that IRE1α RNase activity towards its splicing target *Xbp1* mRNA occurs late after GR, precluding a homeostatic role. Instead, IRE1α activity during GR drives neuronal death by positively regulating ASK1/JNK activity through the degradation of *14-3-3 θ* mRNA, a negative regulator of ASK and an adaptor protein highly expressed in brain, implicated in neuroprotection. Collectively, results describe a novel regulatory mechanism of cell death in neurons, triggered by the downregulation of *14-3-3 θ* mRNA induced by the IRE1α branch of the UPR.

## Introduction

The brain is a highly energy-demanding organ that depends on glucose as the main fuel for correct functioning. Decreased glucose blood levels or disruption of glucose supply to the brain results in neuronal malfunction. Hypoglycemia is the main complication of insulin treatment in type I diabetic patients with strict glycemic control. Severe hypoglycemia occurs when blood glucose concentration drops below 40 mg/dl and can culminate in the hypoglycemic coma, resulting in irreversible brain damage in vulnerable regions such as the cortex, the hippocampus, and the striatum^1,2^. Accumulating evidence suggests that mechanisms triggered during hypoglycemia contribute to delayed neuronal death including oxidative stress, PARP activation, and autophagy^3–5^. However, the role of the unfolded protein response (UPR) in hypoglycemic brain injury has not been described.

The UPR is an adaptive mechanism in response to any disturbance in the endoplasmic reticulum (ER) homeostasis. Activation of the UPR mitigates protein misfolding, as it attenuates protein synthesis, enhances protein degradation, and upregulates target genes involved in proteostasis restoration^6,7^. Three ER-resident transmembrane proteins orchestrate the UPR: inositol-requiring enzyme 1 (IRE1), activating transcription factor 6 (ATF6), and protein kinase RNA (PKR)-like ER kinase (PERK).

Sustained activation of the UPR is implicated in the pathology of several neurodegenerative diseases^8^. PERK activation leads to the blockade of global protein synthesis^9^ and the upregulation of genes involved in the antioxidant defense, autophagy, ER protein folding, and degradation^10^ through the selective translation of the activation transcription factor 4 (ATF4). However, prolonged blockade of protein translation leads to the upregulation of the transcription factor C/EBP homologous protein (CHOP), which promotes the expression of pro-apoptotic genes^11,12^. IRE1 is a transmembrane protein containing a kinase and an endoribonuclease (RNase) domain. IRE1 oligomerization and *trans*-autophosphorylation activate its RNase domain to catalyze the cleavage of the X-binding protein 1 (*Xbp1*) mRNA, generating the active transcription factor XBP1s^13^, which promotes cell survival. In addition, the RNase domain of IRE1 mediates the cleavage of multiple RNAs in a process known as regulated IRE1-dependent decay (RIDD)^14,15^, which can result in apoptosis through the downregulation of mRNAs encoding key targets for protein folding as GRP78^15^. Finally, the IRE1 kinase domain leads to apoptosis through ASK1/JNK signaling by the inactivation of anti-apoptotic BCL2 proteins. Hence, the role of IRE1 in UPR-induced apoptosis is well-known, however, the regulation of UPR targets involved in neurodegeneration has not been completely elucidated.

The 14-3-3 family of adapter proteins are highly expressed in the brain and participate in the regulation of several processes, including signal transduction, neuronal differentiation, migration, and survival, through their capability of binding to a vast number of proteins partners^16,17^. In mammals, this family includes seven isoforms (*β*, *γ*, *ε*, *η*, *ζ*, *σ*, and *θ*) which play an essential anti-apoptotic role by interacting with various pro-apoptotic proteins, including BAD, BAX, and ASK1^18–20^. 14-3-3 proteins have been implicated in several neurodegenerative diseases and their induction during ischemia can reduce apoptotic neuronal death^21^. However, little is known about the regulation of 14-3-3 proteins under ER-stress conditions.

Evidence suggests that altered proteostasis is associated with cognitive dysfunction^22^, neurodegenerative diseases^23^, and ischemic neuronal death^24^. During these conditions, the UPR can either have beneficial or detrimental effects depending on the degree and duration of ER stress^25^. Previous reports from our group demonstrated that the ER-stress inducible caspase-12 is activated during GD in hippocampal neurons and participate in neuronal death^26^. However, the time course activation of the PERK and the IRE1α branches of the UPR during GD and GR is still not clear. Therefore, we aimed to analyze the dynamics of PERK and IRE1α activation in response to GD and GR in cortical neurons, dissect the downstream pathways and their contribution to neuronal demise. Results show that early PERK activation contributes to neuronal adaptation to GD through the inhibition of global protein synthesis and ATF4 induction. However, sustained blockade of global protein translation during GR contributes to neuronal death. An early and sustained IRE1α activity was observed during GD and GR, mediated by its interaction with BAX/BAK and ASK1. Processing of *Xbp1* mRNA by IRE1α occurs late after GR making its homeostatic role unlikely. Instead, activation of IRE1α RIDD activity drives neuronal death by positively regulating ASK1/JNK signaling through the degradation of the mRNA of *14-3-3 θ* isoform, a negative regulator of ASK1^20^. These results reveal a novel regulatory mechanism of 14-3-3 *θ* protein by IRE1α RIDD activity, which drives neuronal death during glucose deprivation.

## Results

### Dynamics of activation of the PERK and IRE1α branches of the UPR

**Fig. 1:**
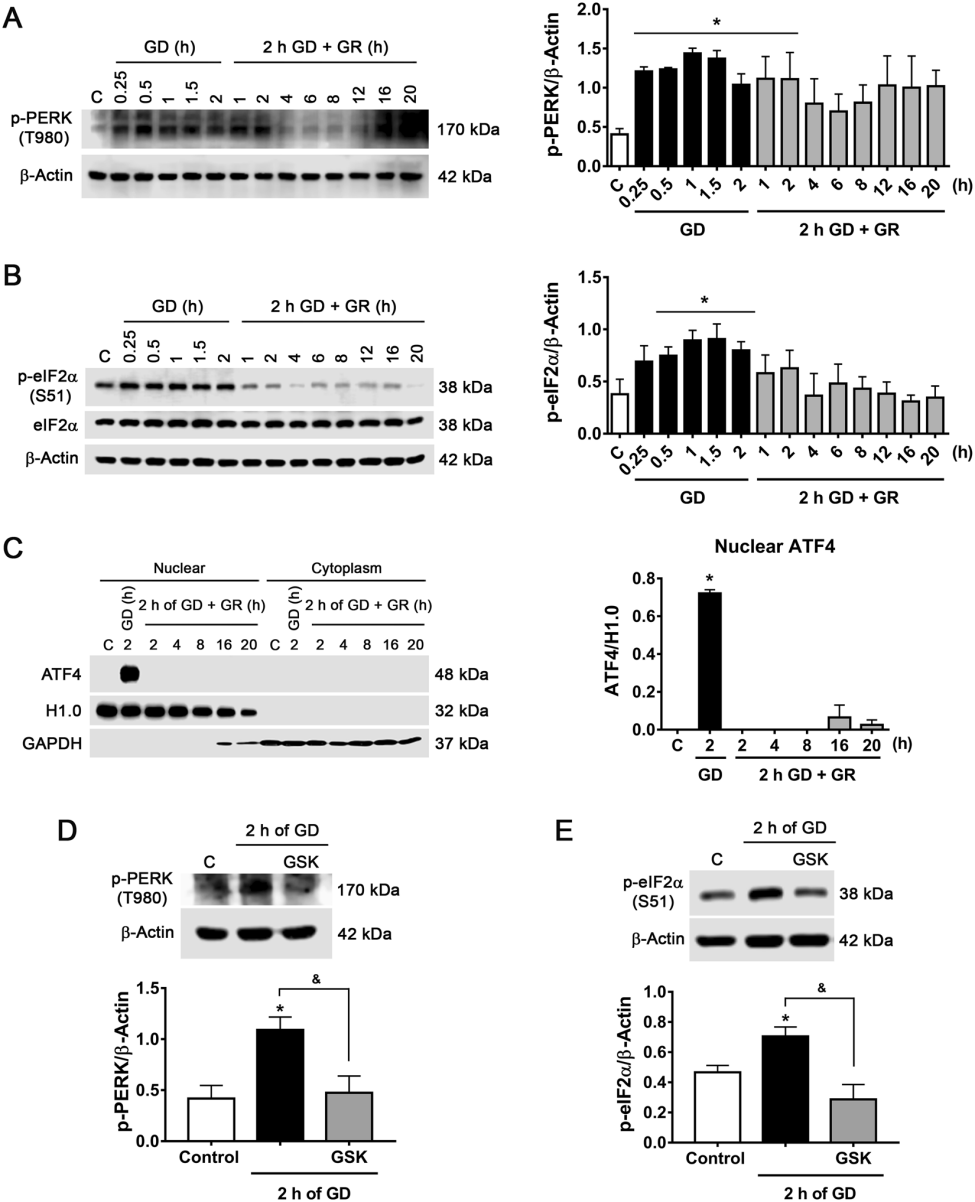
Glucose deprivation induces PERK activation in cortical neurons. **A** Representative immunoblot and quantification of p-PERK/β-Actin. **B** Representative immunoblot and quantification of p-eIF2α/β-Actin. **C** Analysis of AFT4 by subcellular localization. Representative immunoblot and quantification of ATF4 in the nuclear fraction. H1.0 (histone H1) was used as a nuclear protein control and GAPDH as a cytoplasmic protein control. **D** p-PERK levels during GD in the presence or absence of PERK inhibitor GSK (10 µM). Representative immunoblot and quantification of p-PERK/β-Actin. **E** p-eIF2α levels during GD in the presence or absence of GSK. Representative immunoblot and quantification of p-eIF2α/β-Actin. Data represent the mean ± SEM of 3 (**A**, **B**, **D**) and 4 (**C** and **E**) independent experiments and were analyzed by one-way ANOVA followed by Fisher´s multiple comparisons, *p < 0.05 vs. control, ^&^*p* < 0.05 vs. 2 h GD.

The dynamics of activation of PERK and IRE1α during the GD and GR periods were first examined. PERK activation was assessed by measuring its phosphorylation at T980 and that of its target eIF2α at S51, which results in the global block of protein synthesis and the selective translation of the transcription factor ATF4^12^. GD induced a rapid increase in p-PERK and p-eIF2α, which progressively decreased to levels not different from control during GR (Fig. 1A, B). The total content of eIF2α did not change during the GD and GR periods (Fig. 1B). Translation of ATF4 and its translocation to the nucleus was observed 2 h after glucose withdrawal but not during GR, suggesting it is only transiently expressed (Fig. 1C). PERK kinase is part of the integrated stress response, a group of kinases capable to phosphorylate eIF2α^27^. To confirm that increased p-eIF2α is mediated by PERK activity the specific PERK inhibitor GSK 2606414 was used^28^. Neurons treated with GSK during 2 h GD significantly reduced p-PERK and p-eIF2α levels (Fig. 1D, E), confirming eIF2α/ATF4 signaling by PERK activation during GD.

**Fig. 2:**
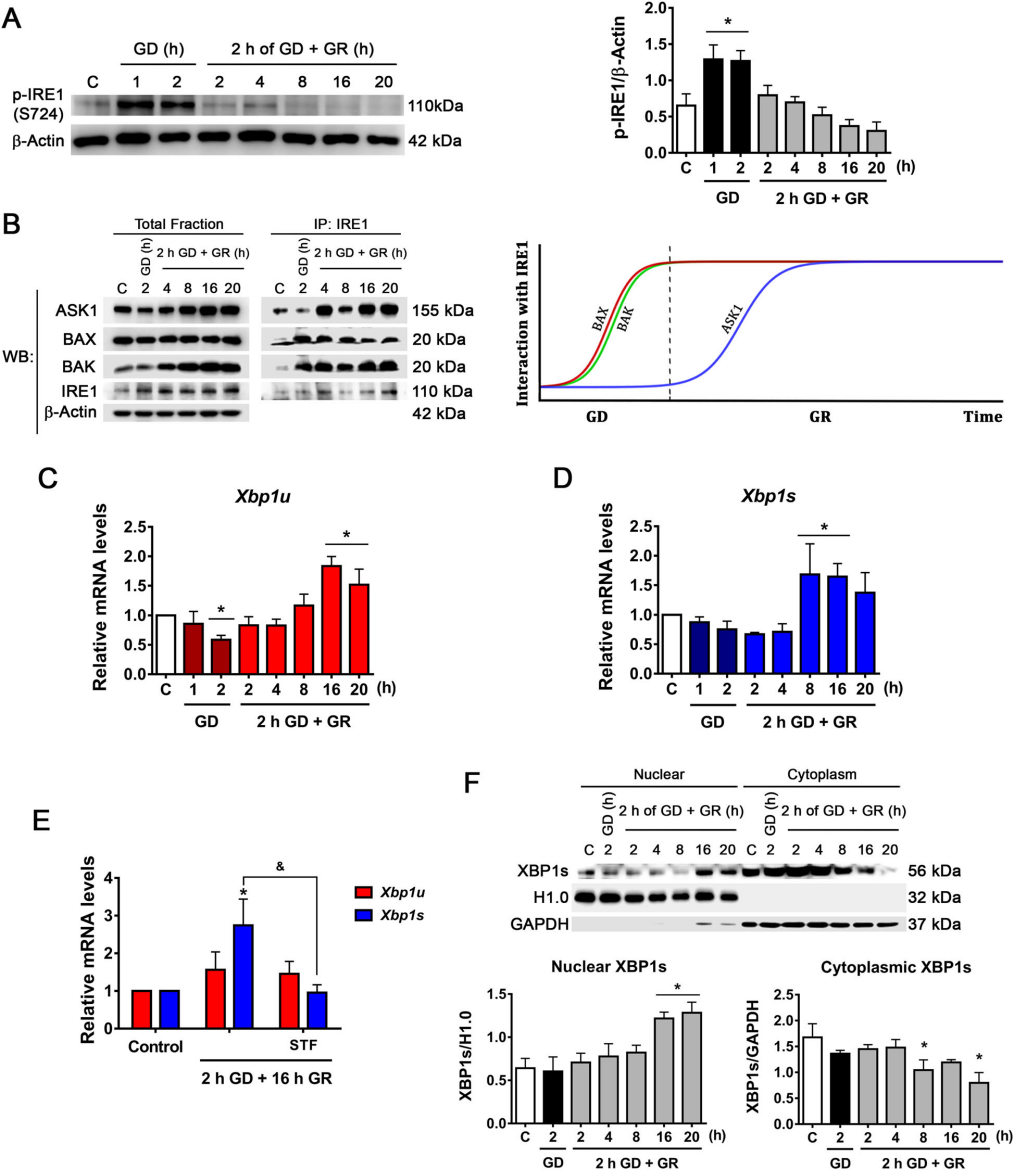
IRE1α signaling activation during GD and GR. **A** Representative immunoblot and quantification of p-IRE1α/β-Actin. **B** Analysis of the interaction of BAK, BAX, and ASK1 with IRE1α by co-immunoprecipitation and detected by immunoblot. Representative immunoblots from two independent experiments and a diagram of the interaction kinetics of BAX, BAK, and ASK1 with IRE1α along the GD and GR periods. Expression levels of **C** *Xbp1u* and **D** *Xbp1s* mRNA levels as determined by qRT-PCR in cortical neurons exposed to GD/GR. **E** Effect of the inhibition of IRE1α RNAse activity on *Xbp1u* and *Xbp1s* expression. IRE1α RNase activity inhibitor (STF, 30 µM) was incubated during GR. **F** Analysis of the subcellular localization of processed XBP1 (XBP1s). Representative immunoblot and quantification of XBP1s in the nucleus and cytoplasm. Data represent the mean ± SEM of 5 (**A**), 4 (**C**, **D**), and 3 (**E**, **F**) independent experiments and were analyzed by one way ANOVA followed by Fisher’s multiple comparisons, *p < 0.05 vs. control, ^&^*p* < 0.05 vs. 2 h GD + 16 h GR.

Accumulation of unfolded proteins induces IRE1α oligomerization and *trans*-phosphorylation, leading to RNase domain activation. We evaluated the phosphorylation of IRE1α at S724^29^ and observed a rapid increase of p-IRE1α upon GD. During glucose replenishment p-IRE1α progressively decreased (Fig. 2A). It is known that several proteins interact with IRE1α and modulate its activity, hence we decided to investigate the IRE1α interactome in the GD/GR model. Immunoprecipitation experiments revealed that the pro-apoptotic proteins BAX and BAK, essential for IRE1α activation during ER stress^30^, associate with IRE1α during GD, and this interaction is maintained throughout the GR period (Fig. 2B). On the other hand, ASK1 interacts with IRE1α through the adapter protein TRAF2 allowing the activation of the JNK pathway^31^. Results revealed that ASK1 also interacts with IRE1α but only during GR (Fig. 2B).

The interaction of BAK/BAX triggers IRE1α RNase activity and enables the splicing of *Xbp1* mRNA^30^. Therefore, we followed the processing of *Xbp1* by qRT-PCR and immunoblot throughout GD and GR. For *Xbp*1 mRNA processing specific primers for the unspliced (*Xbp1u*) and the spliced variants (*Xbp1s*) we designed. Surprisingly, *Xbp1s* levels significantly increased during the late stages of GR (8–16 h) and not earlier (Fig. 2C, D). In addition, primers designed to amplify both mRNA variants were used and resolved in agarose gel. Results show that *Xbp1* mRNA processing occurs late after GR (Fig. S1A). To confirm that *Xbp1* processing is mediated by IRE1α activity, we used the specific IRE1α RNase activity inhibitor, STF-083010^32^. STF added during GR significantly decreased *Xbp1s* levels (Fig. 2E and Fig. S1A), confirming that *Xbp1* mRNA is processed by IRE1α. In agreement, XBP1s protein significantly increased in the nuclear fraction late after GR (Fig. 2F). Accordingly, GRP78, a target gene of this transcription factor also increased after 16 and 20 h of GR (Fig. S1B), suggesting that IRE1α RNase activity remains late after GR. Altogether, these results show the rapid activation of the PERK and IRE1α branches of the UPR in response to GD. However, only IRE1α downstream signaling persists at late stages of GR.

### PERK activation during GD contributes to neuronal survival and sustained activation of IRE1α during GR triggers neuronal death

**Fig. 3:**
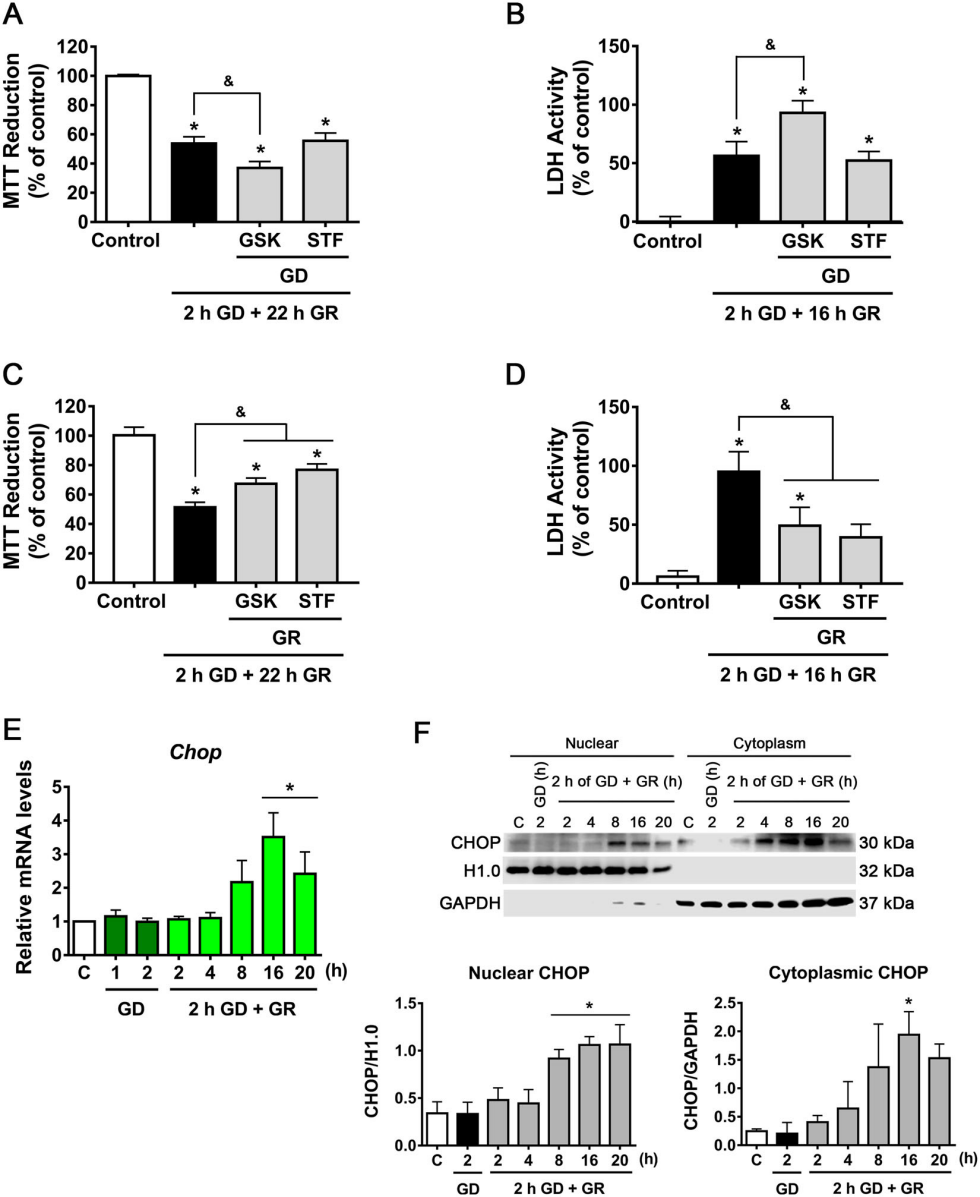
UPR activation contributes to neuronal death. MTT reduction (**A**, **C**) and LDH activity (**B**, **D**) in cortical neurons exposed to GD/GR, in the presence or absence of PERK (GSK, 10 µM) or IRE1 inhibitors (STF, 30 µM), incubated during GD (**A**, **B**) or during GR (**C**, **D**). **E** Expression of *Chop* mRNA in neurons exposed to GD/GR. **F** Subcellular localization of CHOP protein in neurons exposed to GD/GR. Representative immunoblot and quantifications of CHOP in the nucleus. Data represent the mean ± SEM of 5 (**A**–**D**), 4 (**E**), and 3 (**F**) independent experiments and were analyzed by one way ANOVA followed by Fisher’s multiple comparison test, *p < 0.05 vs. control, ^&^*p* < 0.05 vs. 2 h GD + 22 h GR.

To investigate the role of PERK and IRE1α on neuronal survival or delayed neuronal death, cell viability was measured by the MTT reduction and the LDH activity assays. PERK activity inhibition by GSK during GD aggravated cell death, while IRE1α inhibition by STF had no effect (Fig. 3A, B). In contrast, PERK inhibition during GR partially improved neuronal viability while blockade of IRE1α RNase activity by STF increased neuronal survival (Fig. 3C, D).

The transcription factor CHOP is a common ER-stress-induced apoptosis protein regulated by the three branches of the UPR^33^. Hence, we followed *Chop* mRNA levels throughout GD/GR and observed a significant increase at the late stage of GR (Fig. 3E). Accordingly, nuclear enriched fractions showed increased CHOP protein content from 8 to 20 h after GR (Fig. 3F). Hence, the activity of caspases was followed. No change in caspase-3 activation was found (Fig. S2A) suggesting that it is not involved in neuronal death. However, the caspase-12 active fragment, which has been proposed as a key mediator of ER stress-induced apoptosis, was generated soon after glucose withdrawal and remained increased during GR (Fig. S2B). Caspase-12 specific inhibitor QATAD incubated during GR improved cell survival (Fig. S2C), suggesting that its activation during this period contributes to delayed neuronal death. Together these results suggest that early activation of PERK during glucose withdrawal plays an adaptive role in promoting cell survival, while PERK and IRE1α activation during glucose replenishment contributes to delayed neuronal death correlating with increased *Chop* expression and sustained caspase-12 activity.

### Sustained blockade of protein synthesis by PERK activity contributes to delayed neuronal death

**Fig. 4:**
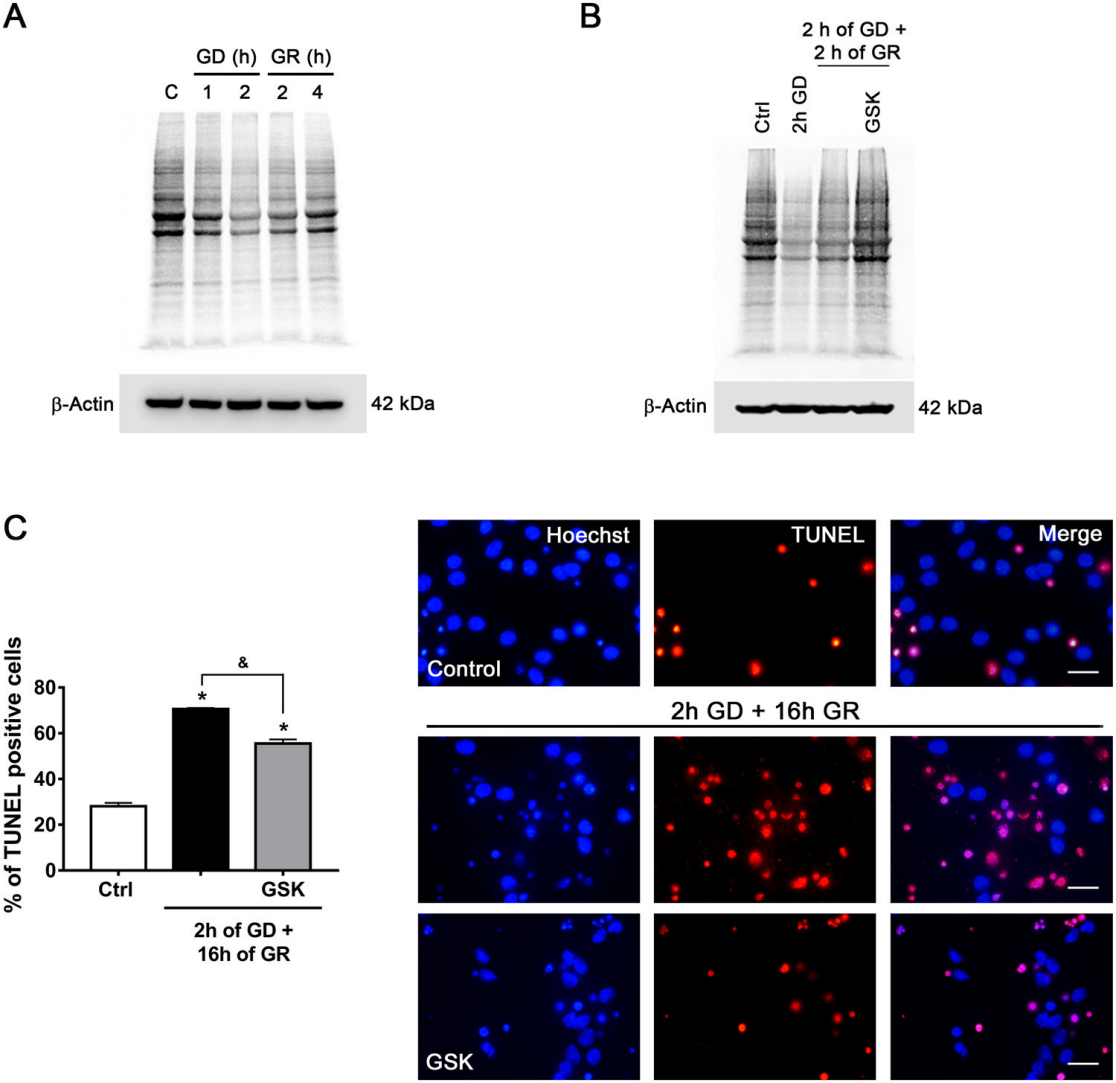
Protein synthesis inhibition by PERK activity during GR promotes apoptosis. **A** Time lapse of ^35^S-methionine incorporation to new polypeptides during GD/GR in cortical neurons. A representative autoradiograph of ^35^S-methionine incorporation to new polypeptides resolved by SDS-PAGE. **B** Effect of PERK inhibition during GR on protein synthesis in cortical neurons. A representative autoradiograph of ^35^S-methionine incorporation to new polypeptides in neurons treated with PERK inhibitor (GSK, 10 µM), drug treatment was only during GR. **C** Quantification and representative micrographs of TUNEL-positive cells exposed to 2 h of GD and 16 h of GR in the presence or absence of PERK inhibitor (GSK, 10 µM). The drug was administered only in the GR phase. Data represent the mean ± SEM of three independent experiments and were analyzed by one-way ANOVA followed by Fisher’s multiple comparison test, *p < 0.05 vs. control, ^&^*p* < 0.05 vs. 2 h GD + 16 h GR. Scale bar = 20 µm.

As it was observed that PERK inhibition during GR confers neuroprotection against delayed neuronal death, we ought to investigate whether the attenuation of the rate of protein synthesis induced by PERK contributes to neuronal survival. First, using pulse and chase experiments, we monitored protein synthesis by measuring the incorporation of radioactive ^35^S-methionine to new polypeptides. We observed that protein synthesis rapidly diminished after 1 and 2 h of GD and persisted partially inhibited from 2 to 4 h after GR as compared to the control condition (Fig. 4A); when PERK is inhibited by GSK during GR, we observed that ^35^S-methionine incorporation was completely recovered at 2 h after GR (Fig. 4B), which correlates with a decrease in terminal deoxynucleotidyl transferase dUTP nick end labeling (TUNEL)-positive cells (Fig. 4C), suggesting that blockade of protein synthesis during GR has a damaging effect and that the restoration of protein synthesis improves neuronal survival.

### IRE1α RIDD activity drives ASK1 activation through the degradation of 14-3-3 θ mRNA

The results described above suggest that IRE1α RNase activity inhibition during GR improves neuronal survival, hence we investigated whether IRE1α RIDD activity has a role, as it has been suggested as the pro-apoptotic output of IRE1α signaling^34–36^. Thus, we searched in the literature for putative RIDD mRNAs substrates, which can be associated with neuronal death. In previous work, the YWHAQ gene was found as a possible target of RIDD activity^37^. The product of this gene is the protein 14-3-3 θ a negative regulator of ASK1^20^. Based on this information it was investigated whether IRE1α RIDD activity stimulates the ASK1/JNK pathway through the downregulation of 14-3-3 θ.

**Fig. 5:**
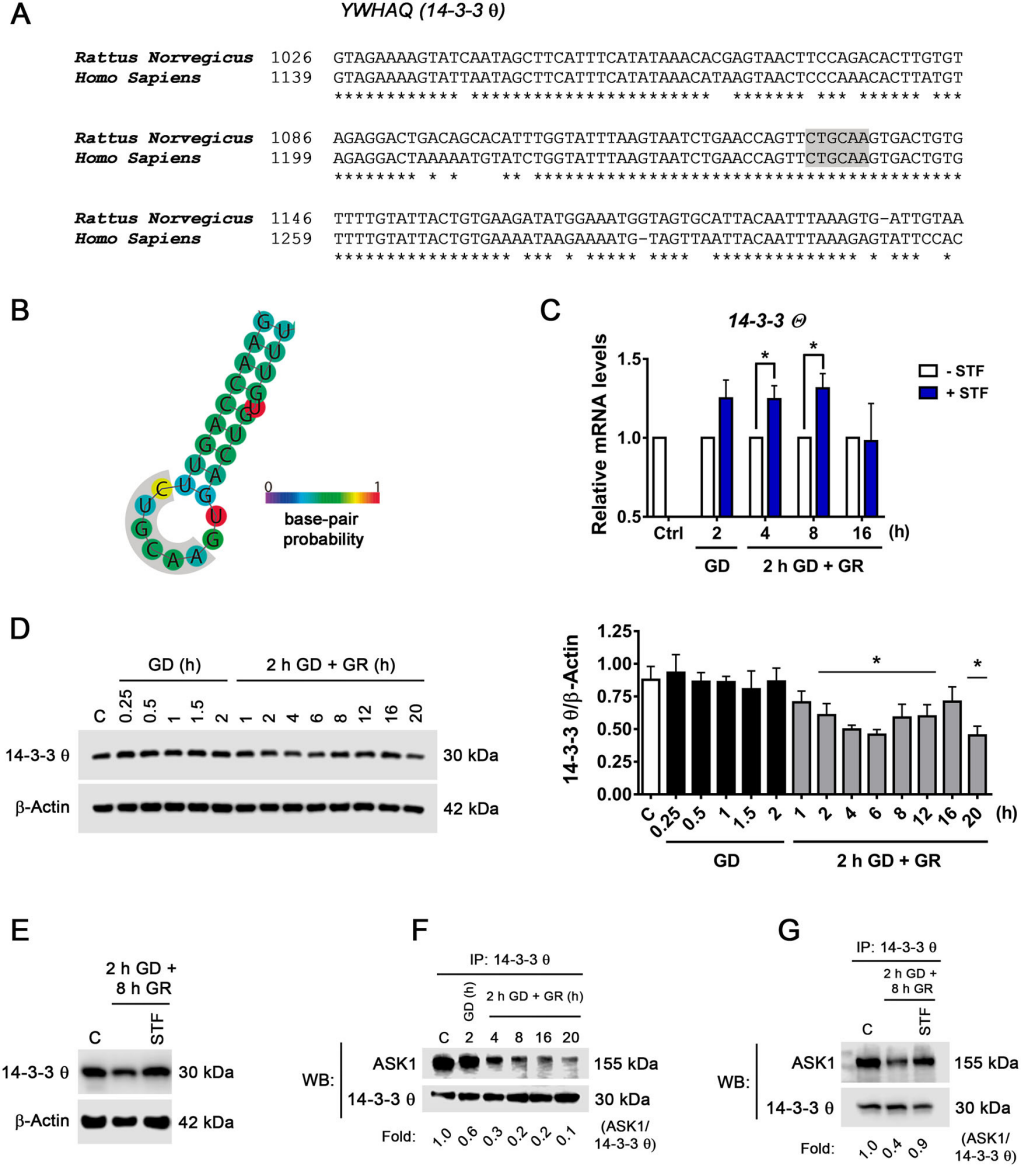
Downregulation of *14-3-3 θ* mRNA by IRE1α RIDD activity stimulates ASK1 activation. **A** Comparison of human and rat *14-3-3 θ* mRNA XBP1 like consensus site (gray). **B** Predicted secondary structure of the *14-3-3 θ* mRNA folded in silico by the RNAfold web server. XBP1 like consensus site (gray area). **C** Effect of the inhibition of IRE1 RNase activity in the expression of *14-3-3 θ* mRNA at different times of GD and GR. STF inhibitor (30 µM) was incubated during GD (2 h) or GR (4, 8, and 16 h). **D** Representative immunoblot and quantification of 14-3-3 θ /β-Actin at different times of GD and GR. **E** Representative immunoblot of 14-3-3 θ /β-Actin the presence or the absence of STF (30 µM) incubated during GR. **F** Analysis of the interaction of 14-3-3 θ with ASK1 by co-immunoprecipitation and detected by immunoblot in neurons exposed to GD and GR. **G** Representative co-immunoprecipitation of 14-3-3 θ and ASK1 in the presence or the absence of STF (30 µM) incubated during GR. Data represent the mean ± SEM of three independent experiments and were analyzed by unpaired Student’s *t* test (**C**) and one-way ANOVA followed by Fisher’s multiple comparison test (**D**), *p < 0.05 vs. control.

RIDD mRNA targets contain a consensus sequence similar to the cleavage site of *Xbp1*, which should be exposed at the loop of a hairpin secondary structure in order to be cleaved. First, we investigated whether the IRE1α restriction site is conserved in the rat *14-3-3 θ* (YWHAQ) gene. Comparing the rat and human DNA sequences 86% homology is observed (Fig. 5A), and specifically, the consensus sequence is conserved (Fig. 5A, Gray). In addition, using the RNAfold Web server, it was found that the ability to form the hairpin structure at the cleavage site is highly likely (Fig. 5B). This in silico analysis suggested that *14-3-3 θ* mRNA is a possible target of IRE1α RIDD activity. Thus, we followed *14-3-3 θ* mRNA levels during GD and at different times after GR, in the presence or the absence of the IRE1α RNase activity inhibitor STF. It was observed that STF increases *14-3-3 θ* mRNA levels after 2 h GD and up to 8 h of GR (Fig. 5C), suggesting that IRE1α RIDD activity can degrade *14-3-3 θ* mRNA. In addition to *14-3-3 θ* mRNA, the effect of STF on the mRNA levels of *Bloc1s1*, another known target of IRE1α RIDD activity, was tested. It was observed that STF also increased *Bloc1s1* mRNA (Fig. S3A), supporting that RIDD activation occurs in the present experimental conditions. The time course of the changes in 14-3-3 θ abundance was also analyzed and as can be observed in Fig. 5D a significant decrease takes place during GR. Furthermore, incubation with STF during GR restored 14-3-3 θ protein content (Fig. 5E) supporting that IRE1α RIDD activity regulates 14-3-3 θ protein abundance.

**Fig. 6:**
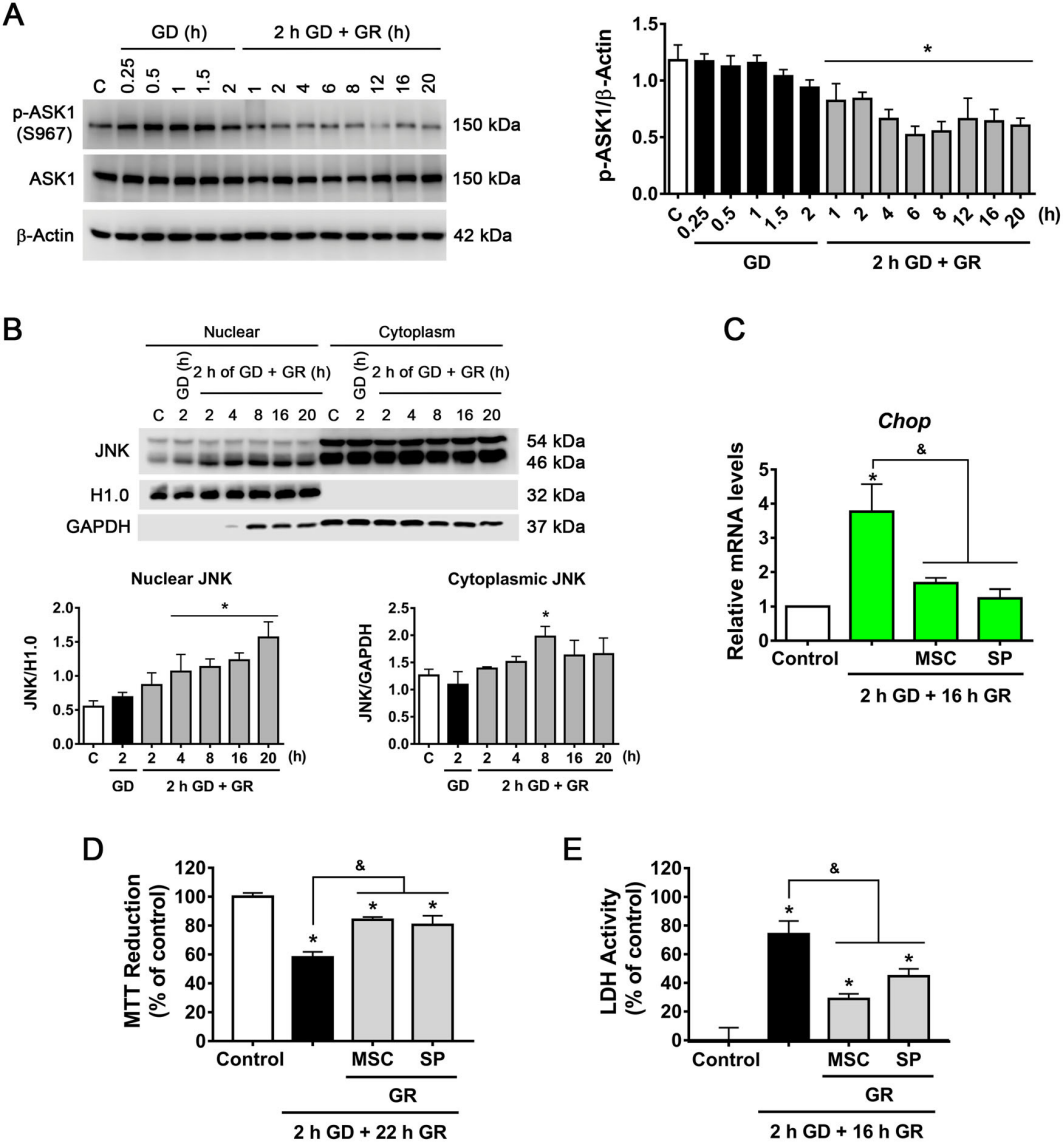
ASK1–JNK contributes to GD/GR-induced neuronal death. **A** Representative immunoblot and quantification of p-ASK1 at different times of GD and GR. **B** Subcellular localization of JNK in cortical neurons exposed to GD/GR. Representative immunoblot and quantifications of JNK (46 kDa isoform) in the nucleus and cytoplasm. **C** Chop mRNA levels in cortical neurons treated with the JNK inhibitor (SP, 10 µM) or ASK1 inhibitor (MSC, 1 µM). **D** MTT Reduction and **E** LDH activity in cortical neurons exposed to GD/GR, in the presence or absence of the ASK1 (MSC, 1 µM) or the JNK inhibitors (SP, 10 µM) during GR. Data represent the mean ± SEM of three independent experiments and were analyzed by one way ANOVA followed by Fisher’s multiple comparison test, *p < 0.05 vs. control, ^&^*p* < 0.05 vs. 2 h GD + 16 h GR (**C**, **E**) or vs. 2 h GD + 22 h GR (**D**).

Time course immunoprecipitation assays revealed that ASK1 progressively loses its interaction with 14-3-3 θ during GR (Fig. 5F) and incubation with STF restored this interaction (Fig. 5G). In addition, the activation of ASK1 was assessed by its phosphorylation at S967. This phosphorylated residue is essential for ASK1 association with 14-3-3 θ and suppression of its activity^38^. We observed a progressive decrease of this phosphorylation site during GR (Fig. 6A), clearly correlating with the loss of ASK1 interaction with 14-3-3 θ. Altogether, these results support the idea that IRE1α RIDD activity degrades *14-3-3 θ* mRNA levels promoting ASK1 activation, which might favor JNK activation during glucose replenishment.

### ASK1–JNK activity is involved in neuronal death

Under ER stress conditions the downstream activation of JNK by IRE1α signaling might amplify ER-stress apoptotic cell death^14^. According to the above-described results, ASK1 is activated after its dissociation from its negative regulator protein 14-3-3 θ, hence we sought to investigate whether the phosphorylation of its main target JNK, is promoted. Three different genes encode JNK proteins producing different variants. Using a pan-antibody, we observed that JNK variants distributed predominantly in two bands of 46 and 54 kDa, respectively. Both variants of p-JNK significantly increased early during GD and at the late stage of GR, suggesting its activation (Fig. S4A).

Previous studies have reported that JNK enters the nucleus and interacts with promoters of cell death-associated genes inducing their expression^39^. Therefore we investigated whether JNK is translocated to the nucleus in the present conditions and whether this is involved in neuronal damage. Subcellular fractionation revealed that the content of the 46 kDa JNK variant increases in the nucleus from 4 to 20 h after GR (Fig. 6B). It has been reported that JNK can regulate *Chop* levels^40,41^, hence we tested whether JNK activity influences CHOP transcription. Bioinformatic analysis of the *Chop* locus was performed with ChIP-seq data against JNK of differentiated glutamatergic neurons (NCBI GEO database). Using the Genome Data Viewer program, it was observed that JNK is present in the promoter of the *Chop* gene (Fig. S5A), suggesting it is able to regulate its expression. When the JNK inhibitor, SP600125, was added during GR, a substantial decrease in *Chop* mRNA levels was observed (Fig. 6C); in addition, the ASK1 inhibitor, MSC also decreased mRNA *Chop* levels (Fig. 6C) supporting the idea that the ASK1/JNK pathway is involved in the upregulation of *Chop*. Cell viability was evaluated in presence of these inhibitors during GR and a clear protective effect was observed (Fig. 6D, E). Together, these data support that activation of the ASK1/JNK pathway is a mediator of IRE1α apoptotic output involved in delayed neuronal death through the up-regulation of *Chop*.

### IRE1α RIDD activity regulates ASK1/JNK activity contributing to apoptotic neuronal death

**Fig. 7:**
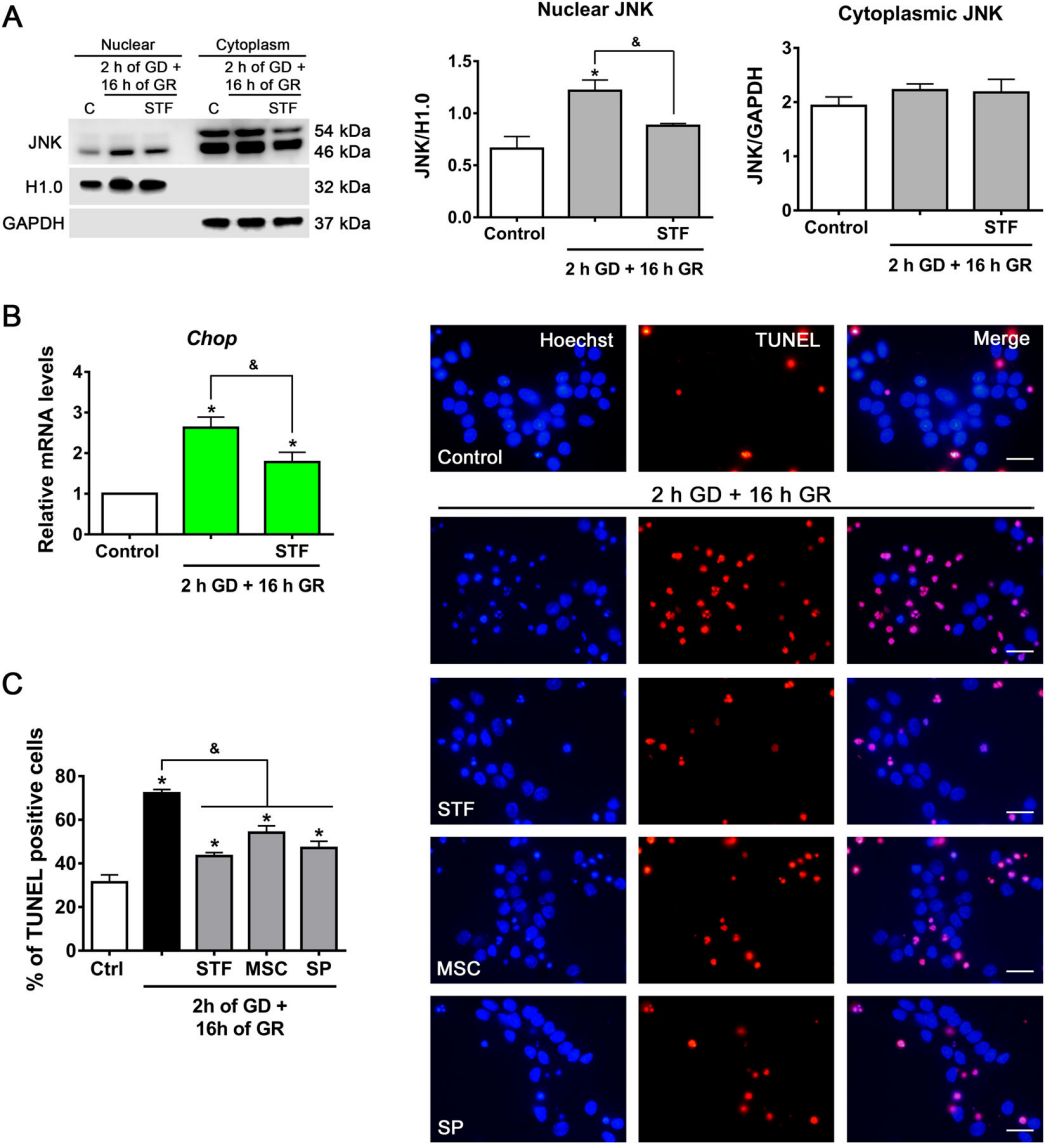
IRE1α RNase activity regulates the ASK1–JNK pathway and induces apoptotic neuronal damage. **A** Subcellular localization of JNK in the presence or absence of the IRE1 RNase inhibitor (STF, 30 µM) incubated during GR. Representative immunoblot and quantifications of JNK in the nucleus and cytoplasm. **B** *Chop* expression in cortical neurons exposed to GD/GR in the presence or absence of IRE1 RNase inhibitor (STF, 30 µM) incubated during GR. **C** Quantification and representative micrographs of TUNEL-positive cells exposed to 2 h of GD and 16 h of GR in the presence or absence of IRE1 RNase (STF, 30 µM), ASK1 (MSC, 1 µM), and JNK (SP, 10 µM) inhibitors. The drugs were administered only in the GR phase. Data represent the mean ± SEM of 3 (**A**, **B**) and 4 (**C**) independent experiments and were analyzed by one way ANOVA followed by Fisher’s multiple comparison test, *p < 0.05 vs. control, ^&^*p* < 0.05 vs. 2 h GD + 16 h GR. Scale bar = 20 µm.

The previously described results suggest that IRE1α RIDD activity downregulates *14-3-3 θ* mRNA, hence it was investigated whether inhibition of IRE1α RNase activity is able to attenuate the activation of the ASK1/JNK pathway. As shown in Fig. 7A, the translocation of JNK to the nucleus is dependent on IRE1α activity, as it is inhibited by STF addition during GR. Furthermore, the increase in *Chop* mRNA was reduced by IRE1α inhibition (Fig. 7B). Finally, the percentage of TUNEL-positive apoptotic cells was reduced by IRE1α, ASK1, and JNK inhibitors (Fig. 7C), suggesting that IRE1α RNase activity drives neuronal death by positively regulating ASK1/JNK activation, and *Chop* expression.

## Discussion

The present study provides evidence of the time-course activation of the PERK and the IRE1α branches of the UPR and their respective contribution to neuronal adaptation or death, in response to energy depletion and recovery in cortical cultured neurons. Results show that PERK activation can be adaptive or deleterious depending on the duration of its activity. Most importantly, results demonstrate for the first time that persistent activation of IRE1α drives neuronal death through downregulation of *14-3-3 θ* mRNA by IRE1α RIDD activity, which positively regulates the ASK1/JNK pathway.

### Fine-tuning of protein synthesis by PERK is essential for neuronal survival

It is well-known that the PERK/ATF4 pathway is an adaptive response to the loss of proteostasis, however, its sustained activation can upregulate the proapoptotic transcription factor CHOP leading to cell death^10^. In the present conditions, the translocation of ATF4 to the nucleus was observed during the GD period, which, however, did not correlate with the upregulation of *Chop* occurring during late GR, making unlikely its participation on delayed apoptotic neuronal death. Instead, results suggest that blockade of global protein synthesis by PERK activation mainly acts as a pro-survival signal for neuronal adaptation during the GD period. In contrast, protein synthesis inhibition during GR contributed to neuronal death. This result agrees with previous data demonstrating that repression of protein synthesis through PERK/p-eIF2α activity, induced by the accumulation of prion proteins, leads to synaptic dysfunction and ultimately to neuronal loss. In these conditions, restored protein synthesis by PERK inhibition conferred protection^42^. Hence, our results support that fine-tuning of protein synthesis by PERK activity could make a difference between cellular adaptation to stress and neuronal death.

### Cell fate is regulated by the IRE1α interactome pattern

IRE1α plays a critical role in cell fate under ER stress conditions and it is a key component of the switch from homeostasis to apoptosis^7,14^. Here we report the activation of the IRE1α/XBP1 pathway in response to GD/GR. However, IRE1α phosphorylation increased soon after glucose withdrawal while Xbp1 splicing occurred late after GR, suggesting that IRE1α trans-phosphorylation might be unnecessary for IRE1α RNase activity in these conditions, as previously suggested^14^. According to the results, XBP1 splicing is not likely involved in the early homeostatic response to GD/GR, although its contribution to homeostasis restoration at the late stages of recovery cannot be discarded.

Increasing evidence supports that IRE1α activity output is regulated by its interaction with several components of other signaling pathways^43,44^. IRE1α interactome is large and constantly expanding. Studies have demonstrated the interaction of IRE1α with BAX/BAK^30^, ASK1-interacting protein 1 (AIP1)^45^, protein tyrosine phosphatase 1B (PTP1B)^46^, and heat shock protein 72 (HSP72)^47^; all these proteins have shown to be positive modulators of IRE1α RNase activity. The present results show an early interaction of IRE1α with the cofactors BAX/BAK during GD, which persists throughout the GR period and promotes IRE1α RNase activity contributing to delayed neuronal death. On the other hand, the cytosolic domain of activated IRE1α binds to the adapter protein TRAF2, triggering alarm signals like the ASK1/JNK^31^, p38, ERK^48^, and NF-κB pathways^49^. In the present conditions, we observed that IRE1α interacts with ASK1 and activates the ASK1/JNK pathway, which is involved in neuronal death. These observations support the hypothesis that IRE1α activity output is regulated by a complex dynamic of association and dissociation with its cofactors and that the expression pattern of the IRE1α interactome could regulate cell fate.

### IRE1α RIDD activity enhances the ASK1/JNK pathway

*Xbp1* mRNA is the only IRE1α splicing target identified so far, however, it has been shown that numerous types of RNA are substrates of IRE1α RIDD activity due to the presence of an XBP1 like a consensus site^37^. RIDD activity has been proposed to be the apoptotic output of IRE1α RNase activity, however, degradation of specific RIDD targets may impact particular signaling pathways in a cell-type-specific manner^50^. Here, we identified a novel regulatory mechanism of 14-3-3 θ isoform in neurons during energy stress through IRE1α RIDD activity. 14-3-3 proteins are strongly related to the pathogenesis of several neurodegenerative diseases and enhancement of their expression shows robust neuroprotective effects^16,21,51^. Previous studies suggest that overexpression of 14-3-3 zeta can protect hippocampal neurons from ER-stress damage^52^ and specifically, 14-3-3 θ isoform has been shown to be protective in a model of Parkinson’s disease by interacting with a-synuclein^53^. Interestingly, the mRNA consensus site that recognizes IRE1 is only present in 14-3-3 θ isoform and not the others^37^, making this a very specific mechanism of regulation. 14-3-3 θ isoform can also bind proapoptotic proteins such as BAX^19^ and ASK1 promoting cell survival^20,54^. In agreement, our results suggest that *14-3-3 θ* mRNA degradation by IRE1α RNase activity, enhances IRE1α/ASK1/JNK pathway inducing JNK nuclear translocation and CHOP expression.

It has been shown that JNK signaling contributes to cellular death triggered by IRE1α activation under persistent ER-stress^14,55^. JNK translocation to the nucleus has been observed in cardiac cells after ischemic injury^56^, and its activity in the nucleus can be critical for neuronal death^57^. Tiwari et al.^39^ based on Chip-seq data showed that JNK can bind to the CHOP promoter and regulate its expression. The present results agree with these observations and indicate that increased *Chop* mRNA expression during GR is dependent on ASK1 and JNK activity.

Together, the present findings indicate that ER-stress induced by GD/GR activates IRE1α RIDD activity and leads to *14-3-3 θ* mRNA degradation and a subsequent decrease in 14-3-3 θ protein content. This in turn enhances the activity of the ASK1/JNK pathway and increases CHOP expression, which ultimately induces neuronal death. These results provide new information relevant for the understanding of death mechanisms downstream of IRE1α and PERK activation in neurons, which might offer new tools for the control of prolonged reticulum stress associated with glucose limiting conditions such as hypoglycemia and some neurodegenerative diseases.

## Materials and methods

### Cell culturing

Primary neuronal cultures were prepared from rat E17 embryos obtained from pregnant Wistar rats provided by the animal house of Instituto de Fisiología Celular, UNAM. All efforts were made to minimize the number of animals used and their suffering. Animals were handled according to the National Institute of Health Guide for the Care and Use of Laboratory Animals (NIH publications No. 80-23, revised 1996) and with the approval of the Animal Care Committee (CICUAL, LMT160-20) of the Instituto de Fisiología Celular, UNAM. Briefly, the cerebral cortex was dissected and chopped, then incubated with 0.25% trypsin/10% EDTA solution at 37 °C for 3 min; the digestion was stopped with a solution containing 0.52% of soybean trypsin inhibitor and 0.08 % of DNase. Cells were suspended in Neurobasal Medium (Gibco, 21103-049, Grand Island, NY, USA) supplemented with 1% of B27 (Gibco, 17504-044), 1% B27 without antioxidants (Gibco, 10889-038), 0.5 mM l-Glutamine, 20 mg/mL gentamycin (Gibco, 15710-064) and plated at a density of 2.2 × 10^5^ cells/cm^2^ in plates precoated with poly-l-Lysine (Sigma-Aldrich, P-1524, St. Louis MO, USA). Cells were cultured at 37 °C in a humidified 5% CO_2_/95% air atmosphere. Cytosine-d-Arabinoside 0.54 mM (Sigma, C-1768) was added to cultures 4 days after plating.

### Cell treatments

Experiments were carried out at eight DIV. To induce glucose deprivation (GD), the culture medium was removed and changed for DMEM free-glucose medium (Gibco, 11966-025) for different periods of time. After GD the free-glucose medium was replaced with the medium where cells were originally cultivated for different periods of time (Glucose reperfusion period, GR). To determine the role of the UPR main signaling branches in the GD/GR induced damage, cells were treated with the following inhibitors: caspase-12 inhibitor 20 µM (QATAD, MP Biomedicals, 03OPH04801, Solon, OH, USA); IRE1 RNAse inhibitor 30 µM (STF-083010, Sigma-Aldrich, 412510); PERK inhibitor 10 µM (GSK2606414, Sigma-Aldrich, 516535); JNK inhibitor 10 µM (SP600125, Sigma-Aldrich, 420119) and ASK1 inhibitor 1 µM (MSC 2032964A, Tocris, 5641, Bristol, UK). These inhibitors were added during the GD or GR period as described in the “Results” section.

### qRT-PCR

Total RNA was isolated with Trizol reagent (Invitrogen, 15596026, Carlsbad, CA, USA). cDNA was synthesized from 2 µg of RNA with the use of the High Capacity cDNA Reverse Transcription Kit (Applied Biosystems, 4368814, Foster City, CA, USA). Reverse transcription reaction was carried out in a thermal cycler as follows: 25 °C for 10 min, 37 °C for 120 min, and 85 °C for 5 min. For each real-time PCR reaction, 50 ng of cDNA was used.

Primer Sequences of *Xbp1 spliced, Xbp1 unspliced, Chop, 14-3-3 θ, Bloc1s1* and *α-tubulin* were:

*Xbp1 spliced*: 5′-TCAGACTACGTGCGCCTCT-3′, 5′-CTCTGGGGAAGGACATTTGA-3′; *Xbp1 unspliced*: 5′-CTGAGTCCGCAGCAGGTG-3′, 5′-TAGCAGACTCTGGGGAAGGA-3′; *Chop*: 5′-GAAAGCAGAAACCGGTCCAAT-3′, 5′-GGATGAGATATAGGTGCCCCC-3′;

*14-3-3 θ*: 5′-AGGACTGACAGCACATTTGG-3′, 5′-GAAAGGAAACCCCCAAGAAA- 3′;

*Bloc1s1*: 5′-GATTGGCATGGTGGAAAACT- 3′, 5′-ATTCATGGCTTGCCAGTCTC-3′;

*α-Tubulin*: 5′-GATCTGATGTATGCCAAGCG-3′, 5′-TCCACAGAATCCACACCAAC-3′.

### Xbp1 processing analysis

cDNA obtained from cortical cultures was used as a template for PCR amplification. The primers flank the 26 nt introns of the gene Xbp1, the primers used were: 5′-ACACGCTTGGGGATGAATGC-3′ and 5′-CCATGGGAAGATGTTCTGGG-3′. The reaction was carried out in a thermal cycler and the PCR products were run on 3% agarose gel and stained with ethidium bromide as previoulsy reported^58^.

### Protein synthesis assay

Cells were cultured in 35 mm dishes. Before finishing each experimental condition, cells were labeled with 20 µCi of ^35^S-methionine (PerkinElmer, NEG709A, Waltham, MA, USA) for 1 h. Cells were washed with ice-cold 0.1 M phosphate-buffered saline (PBS) and scrapped with lysis buffer (50 mM Tris-HCl pH 8.0, 150 mM NaCl, 1% Triton X-100, 0.5% sodium deoxycholate, and 1% sodium dodecyl sulfate (SDS)) containing 2 mg/mL of protease inhibitor cocktail (cOmplete, Roche, 11836145001 Basel, Switzerland) 30 µg of protein was separated on sodium dodecyl sulfate-polyacrylamide gel electrophoresis (SDS-PAGE), transferred to a PVDF membrane, and analyzed with a Typhoon 8600 phosphorimaging device (GE Healthcare, Chicago, IL, USA).

### MTT cell viability assay

The viability of cortical neurons was measured by the 3-(4, 5-dimethylthiazol-2-yl)-2, 5-diphenyltetrazolium bromide (MTT, Sigma-Aldrich, M2128) reduction assay, which is indicative of viable mitochondria. Cells were exposed for 2 h to GD and 22 h of GR; after 22 h of GR, MTT (60 μg/ml) was added and incubated for 1 h at 37 °C. The resulting formazan salt was dissolved with 2-propanol-HCl and monitored at 570 nm in a spectrophotometer. Data are expressed as a percent of control.

### Lactate dehydrogenase (LDH) activity

Cell viability was also measured by the LDH assay. Cells were exposed to 2 h GD and 16 h after GR, 200 μl of culture medium were collected for LDH activity determination. Samples were incubated with 9.4 mM NADH in 1 mM K_2_HPO_4_/KH_2_PO_4_ buffer for 5 min at room temperature. The reaction was started with 20 mM pyruvate and was followed for 5 min by the decrease in NADH fluorescence at 340 nm using a Beckman Coulter life science UV/vis spectrophotometer. Data were normalized to control values and are expressed as percent activity in the medium relative to control.

### TUNEL

The cells were plated on coverslips and exposed to 2 h GD and 16 h GR in the presence or absence of the drugs. After the treatments, the cells were washed with PBS and fixed for 20 min with 4% paraformaldehyde. Then they were blocked with a solution of 3% H_2_O_2_ in methanol for 10 min at room temperature. After the blockade, the cells were washed with PBS and permeabilized with 0.1% Triton X-100 and 0.1% sodium citrate on ice for 2 min. The TUNEL reaction mixture (In Situ Cell Death Detection Kit, TMR Red; Roche Diagnostics, 12156792910) was added at 37 °C for 1 h, and cells covered from light. Finally, cells were stained with Hoechst 0.001% (Sigma-Aldrich, 33258) in PBS, and the coverslips were mounted on slides. Cells were observed under an epifluorescence microscope (Nikon Eclipse Ci using AT-EGFP/F and AT-DAPI filter) with a 60× objective and nuclei were counted from seven different fields of each experimental condition per individual experiment. Results are expressed as a percentage of TUNEL positive cells with respect to the total number of cells present in each condition. The investigator was blinded to experimental conditions for the quantification of TUNEL-positive cells.

### Subcellular fractionation

Cells were cultured in 60 mm dishes. After treatment cells were washed with ice-cold 0.1 M PBS, suspended in buffer A (Sucrose 0.25 M, 1 mM EDTA, 20 mM HEPES, 10 mM KCl, 1.5 mM MgCl_2_, 0.1% Triton X-100, and 1 mM DTT) with 2 mg/mL of protease inhibitor cocktail (cOmplete, Roche), softly stirred for 10 min a 4 °C and centrifuged at 1000*g* for 10 min. The supernatant fraction was collected as the cytoplasmic extract. The pellet was washed and resuspended in lysis buffer (50 mM Tris-HCl pH 8.0, 150 mM NaCl, 1% Triton X-100, 0.5% sodium deoxycholate, and 1% SDS) with 2 mg/mL of protease inhibitor cocktail (cOmplete, Roche) to obtain the nuclear fraction.

### Immunoblotting

Cells were cultured in 35 mm dishes. After treatment cells were washed with ice-cold 0.1 M PBS and lysed with a lysis buffer (50 mM Tris-HCl pH 8.0, 150 mM NaCl, 1% Triton X-100, 0.5% sodium deoxycholate and 1% SDS) containing 2 mg/mL of protease inhibitor cocktail (cOmplete, Roche). Samples were centrifuged at 2000*g* at 4 °C for 5 min. Protein concentration was determined by Lowry assay and 30 μg of protein from each sample was separated in SDS-PAGE and subsequently transferred to PVDF membranes (Immobilon-P Membrane, Merk Millipore, IPVH00010, Burlington, MA, USA). The membranes were blocked with 5% dry milk in TBS and incubated overnight at 4 °C with specific antibodies against: p-PERK (1:500, Cell Signaling, 3179 S, Danvers, MA, USA); eIF2α (1:2000, Abcam, ab5369, Cambridge, UK); ATF4 (1:1000, Cell Signaling, 11815); p-eIF2α (1:1000, Cell Signaling, 9721); IRE1 (1:1000, Cell Signaling, 3294); p-IRE1 (1:1000, Abcam, ab48187); XBP1 (1:500, Santa Cruz Biotechnology, sc-7160, Dallas, TX, USA); SAPK/JNK (1:1000, Cell Signaling, 9252); p-SAPK/JNK (1:3000, Cell Signaling, 9251); ASK1 (1:1000, Cell Signaling, 8662); p-ASK1 (1:1000, Cell Signaling, 3764); CHOP (1:500, Santa Cruz Biotechnology, sc-7351); BAK (1:1000, Cell Signaling, 2314); BAX (1:1000, Merk Millipore, ABC11); 14-3-3 ϴ (1:8000, Santa Cruz Biotechnology, sc-69720); active Caspase-12 (1:2000, Sigma-Aldrich, PRS3197); active Caspase-3 (1:1000, Cell Signaling, 9661); GRP78 (1:16,000, Abcam, ab21685); β-Actin (1:7000, Merck Millipore, MAB1501); H1.0 (1:1000, Abcam, ab11079-100) and GAPDH (1:16,000, Cell Signaling, 2118).

### Co-immunoprecipitation

After treatment, the cells were washed with cold 0.1 M PBS and resuspended in IP buffer (20 mM Tris-HCl pH 7.5, 1 mM EDTA, 20 mM NaCl, and 1% Triton) with 2 mg/ml protease inhibitor (cOmplete, Roche). Protein was quantified by the Lowry’s method and 200 μg of protein was incubated with 1 µl of IRE1 (Cell Signaling, 3294) or 1 µl of 14-3-3 θ (Santa Cruz Biotechnology, sc-69720) antibody overnight at 4 °C. Subsequently, it was incubated with A-sepharose protein (Sigma-Aldrich, P3391) beads for 2 h at 4 °C. After the incubation, the beads were washed three times with buffer C (0.02 M NaH_2_PO_4_, 0.15 M NaCl, pH 8). The beads were resuspended in Lamelli with 10% B-Mercaptoethanol and heated for 10 min. The supernatant was used as the immunoprecipitate and loaded on SDS-PAGE gels.

### ChIP-seq bioinformatics analysis

ChIP-seq data for JNK used in this study were obtained from GEO (Gene Expression Omnibus) database with the access name GSE25533; results were obtained from the study: A chromatin-modifying function of JNK during stem cell differentiation^39^. The data were visualized in the NCBI Genome Data Viewer program (National Center for Biotechnology Information).

### Statistical analysis

No statistical method was used to calculate the sample size before the study. A minimum sample size of three biological replicates was used in order to minimize the number of pregnant rats used. The data obtained are presented as the mean ± the standard error from at least three independent experiments. Subsequently, they were statistically analyzed using unpaired Student’s *t* test (two-tailed) or one-way ANOVA followed by the Fisher multiple comparison test with a statistical significance of *p* ≤ 0.05 using GraphPad Prism 7 software.

## Supplementary information

Supplementary Figure 1. XBP1 processing in neurons exposed to GD/GR

Supplementary Figure 2. Induction of apoptosis by UPR signaling during GR.

Supplementary Figure 3. Processing of Bloc1s1 mRNA by IRE1 RIDD activity in neurons during GD/GR induced death.

Supplementary Figure 4. Levels p-JNK in neurons exposed to GD/GR.

Supplementary Figure 5. Presence of JNK in the promoter region of Chop locus.
