# Supplementary figures and images for: IRE1α RIDD activity induced under ER stress drives neuronal death by the degradation of 14-3-3 θ mRNA in cortical neurons during glucose deprivation
Source: Cell Death Discov. 2021 Jun 3;7:131. doi: 10.1038/s41420-021-00518-9 (PMC8175356; doi:10.1038/s41420-021-00518-9)

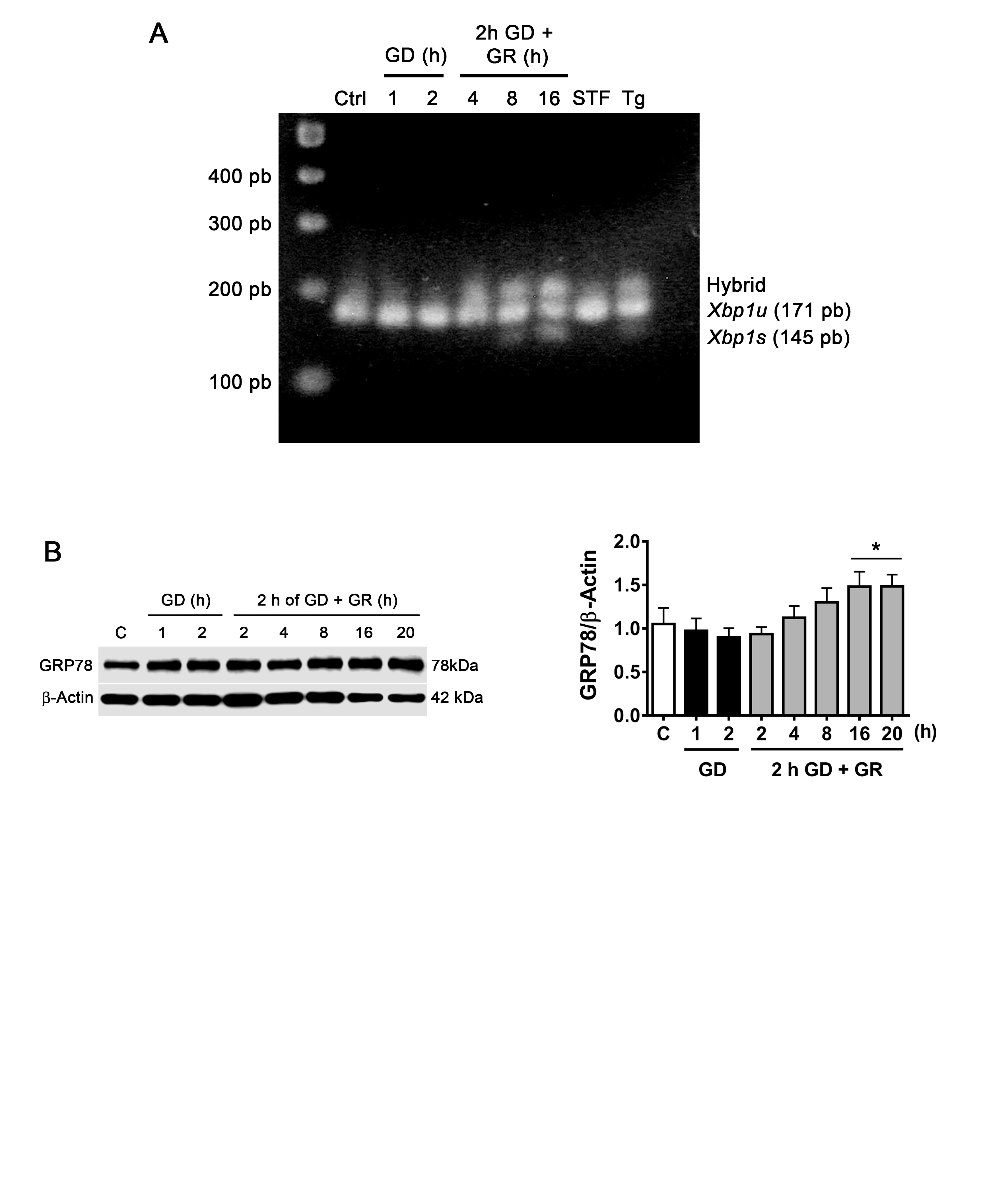

Supplement: Supplementary file 1 — Supplementary Figure 1. XBP1 processing in neurons exposed to GD/GR [file 41420_2021_518_MOESM1_ESM.tif]

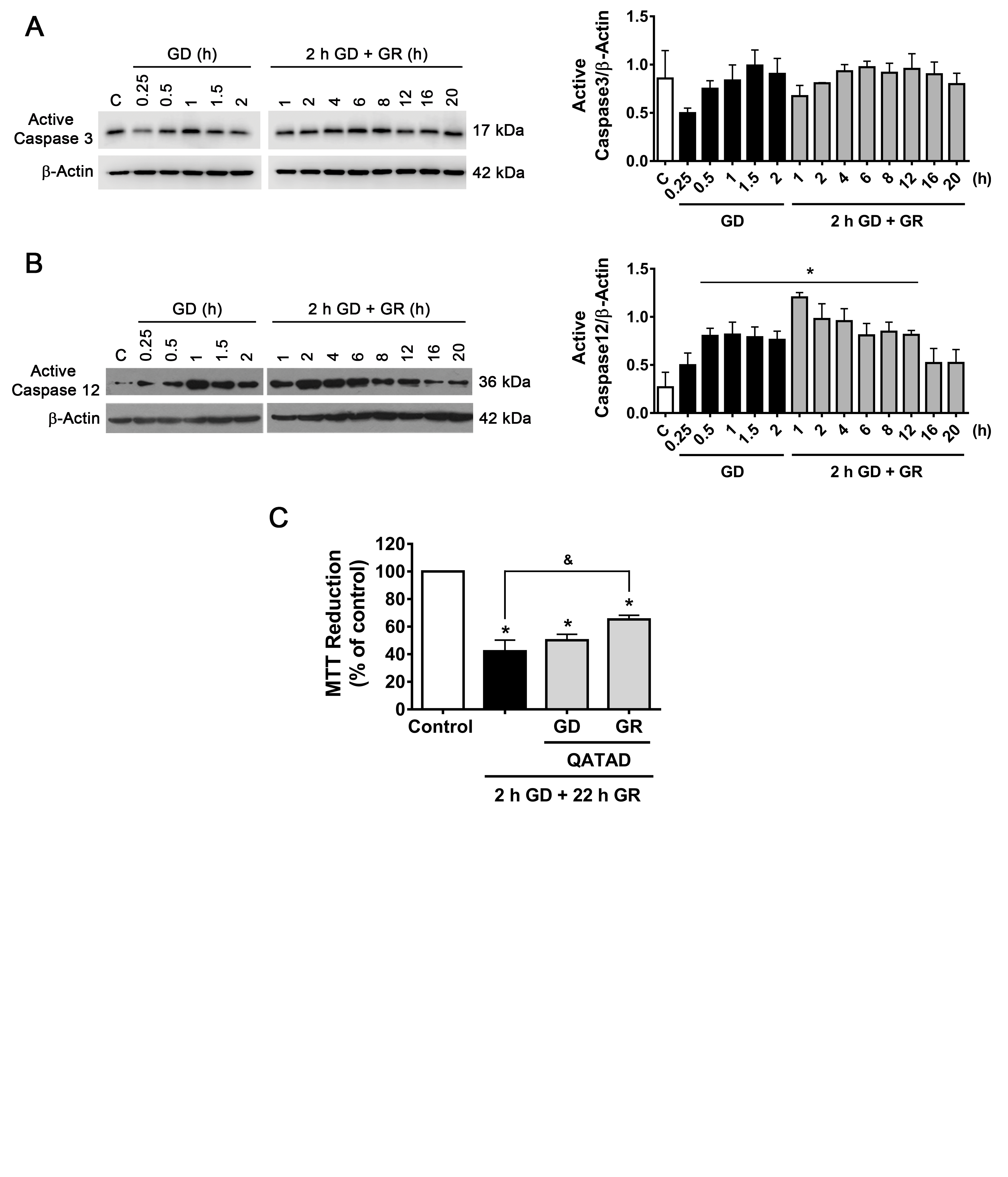

Supplement: Supplementary file 2 — Supplementary Figure 2. Induction of apoptosis by UPR signaling during GR. [file 41420_2021_518_MOESM2_ESM.tif]

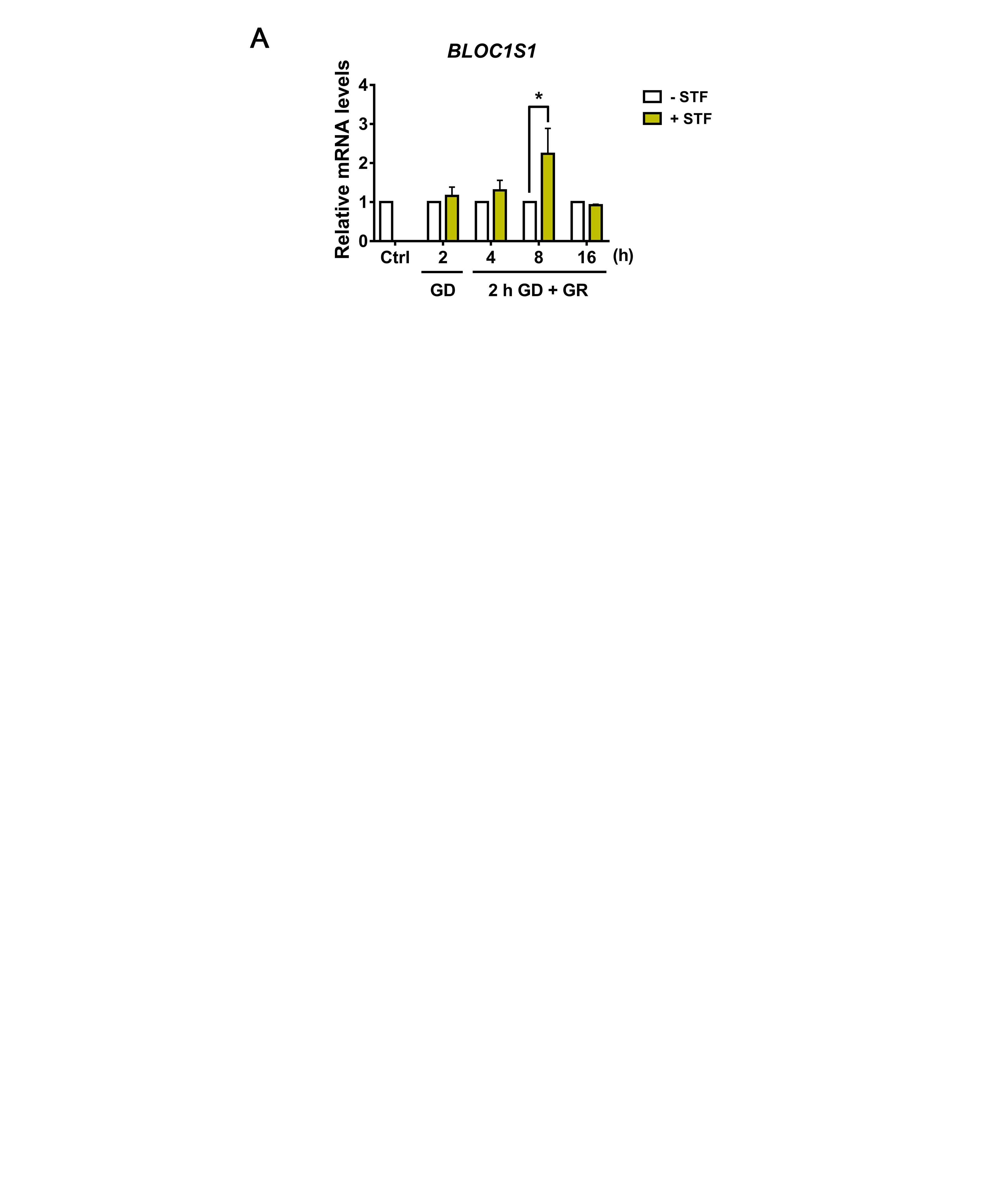

Supplement: Supplementary file 3 — Supplementary Figure 3. Processing of Bloc1s1 mRNA by IRE1 RIDD activity in neurons during GD/GR induced death. [file 41420_2021_518_MOESM3_ESM.tif]

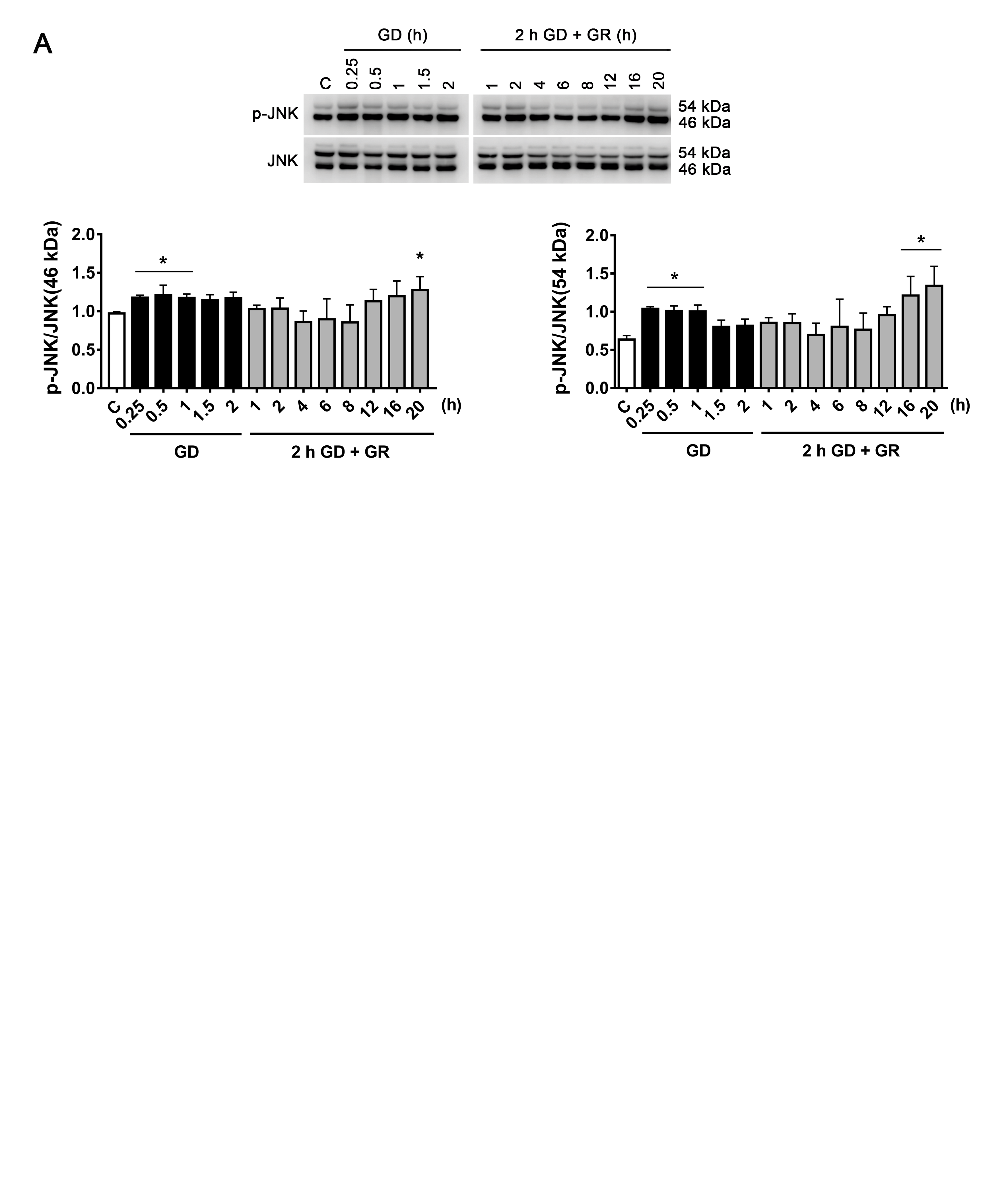

Supplement: Supplementary file 4 — Supplementary Figure 4. Levels p-JNK in neurons exposed to GD/GR. [file 41420_2021_518_MOESM4_ESM.tif]

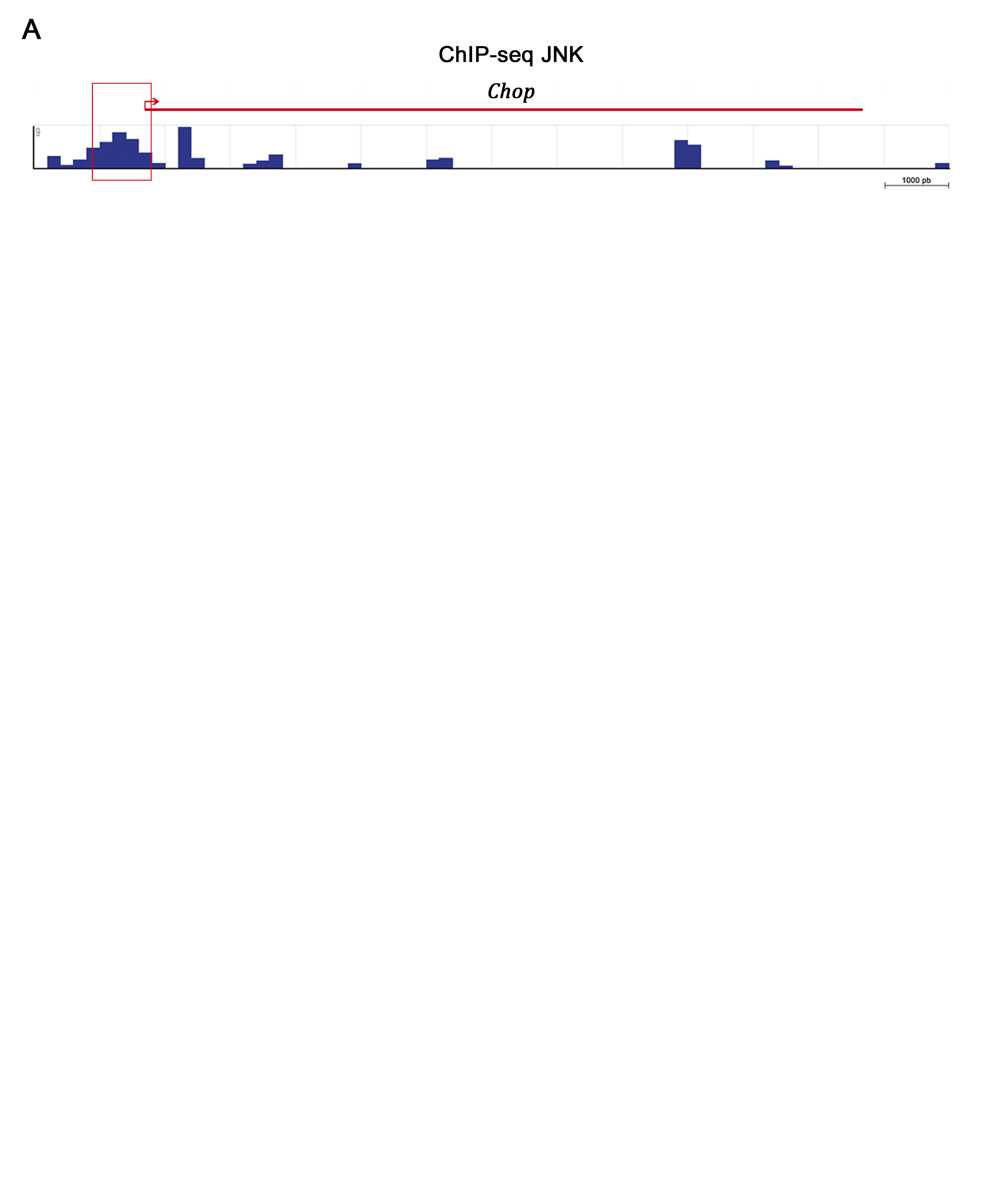

Supplement: Supplementary file 5 — Supplementary Figure 5. Presence of JNK in the promoter region of Chop locus. [file 41420_2021_518_MOESM5_ESM.tif]
